# G-Jitter Induced Magnetohydrodynamics Flow of Nanofluid with Constant Convective Thermal and Solutal Boundary Conditions

**DOI:** 10.1371/journal.pone.0122663

**Published:** 2015-05-01

**Authors:** Mohammed J. Uddin, Waqar A. Khan, Ahmad Izani Md. Ismail

**Affiliations:** 1 Department of Mathematics, American International University-Bangladesh, Banani, Dhaka, Bangladesh; 2 Department of Engineering Sciences, PN Engineering College, National University of Sciences and Technology, Karachi, Pakistan; 3 School of Mathematical Sciences, Universiti Sains Malaysia, Penang, Malaysia; Bascom Palmer Eye Institute, University of Miami School of Medicine, UNITED STATES

## Abstract

Taking into account the effect of constant convective thermal and mass boundary conditions, we present numerical solution of the 2-D laminar g-jitter mixed convective boundary layer flow of water-based nanofluids. The governing transport equations are converted into non-similar equations using suitable transformations, before being solved numerically by an implicit finite difference method with quasi-linearization technique. The skin friction decreases with time, buoyancy ratio, and thermophoresis parameters while it increases with frequency, mixed convection and Brownian motion parameters. Heat transfer rate decreases with time, Brownian motion, thermophoresis and diffusion-convection parameters while it increases with the Reynolds number, frequency, mixed convection, buoyancy ratio and conduction-convection parameters. Mass transfer rate decreases with time, frequency, thermophoresis, conduction-convection parameters while it increases with mixed convection, buoyancy ratio, diffusion-convection and Brownian motion parameters. To the best of our knowledge, this is the first paper on this topic and hence the results are new. We believe that the results will be useful in designing and operating thermal fluids systems for space materials processing. Special cases of the results have been compared with published results and an excellent agreement is found.

## Introduction

The presence of temperature/concentration gradients and gravitational field yield convective flows in non-porous and porous media (Uddin et al. [1]). This type of flow has a significant impact on the homogenous melt growth of semiconductor or metal crystals on earth-bound conditions (Uddin et al. [1]). It is known that in space, the gravity effect is reduced as a result both the thermal buoyancy effect and solutal buoyancy effect are also reduced. The convective flow is suppressed in the presence of microgravity environment. The g-jitter (or residual accelerations) originates from a variety of sources such as crew motions, mechanical vibrations (pumps, motors, excitations of natural frequencies of spacecraft structure), spacecraft maneuvers, atmospheric drag and the earth’s gravity gradient (Li [2]). Many researchers investigated g-jitter convective flow in various aspects (see, for example, Chen and Saghir [3]). The g-jitter effects on viscous fluid flow and porous medium have also been investigated by Rees and Pop [4], Chen and Chen [5]. Shu et al. [6] reported double diffusive convection driven by g-jitter in a microgravity environment. In 2002, the same authors [7] extended their previous work by incorporating external magnetic field. They used a finite element method for computation. Sharidan et al. [8] studied g-jitter free convection flow in the stagnation-point region of a three-dimensional body. Wasu and Rajvanshi [9] studied unsteady mixed convection flow under the influence of gravity modulation and magnetic field. The gravity modulation and magnetic field effect on the unsteady mixed convection flow subject to the influence of internal heating and time-periodic gravity modulation effect on thermal instability in a packed anisotropic porous medium was investigated by Bhadauria et al. [10]. The gravity modulation effects on the free convective flow of elastico-viscous fluid were studied by Dey [11].

Recent interest in this subject has been motivated by development in microelectromechanical systems (MEMS) and nanoelectromechanical systems (NEMS). The devices associated with MEMS/NEMS produce lot of heat, which directly affect the usual performances of the devices and reduces longevity. Therefore, an efficient cooling system is necessary in designing MEMS/NEMS components. Choi [12] has shown that nanofluids can enhance thermal conductivity of the fluid as well as the bounding surface. Momentum, heat and mass transfer relevant to nanofluids flow have received considerable attention of many researchers due to their diverse applications in a number of industrial sectors where heat transfer/mass plays a major role. Some of the applications are reported in a recent paper of Uddin e t al. [13]. So far, two mathematical models are available for boundary layer flow of nanofluids in porous/nonporous media: (i) Buongiorno [14] model and (ii) Tiwari and Das model [15]. The former model involves Brownian motion and thermophoresis effects whilst the latter model involves solid volume fraction as a parameter and can be used to analyze the behavior of nanofluids. These two models have been used by many investigators in various aspects. As an example, Nield and Kuznetsov [16] for porous media, Gorla and Chamkha [17] for non-isothermal effects, Yasin et al. [18] for heat generation effects, Kuznetsov [19] for bioconvection, Murthy et al. [20] for magnetic effect on thermally stratified medium. Nield and Kuznetsov [21] presented, an analytical study of fully-developed laminar forced convection in a parallel-plate channel in porous medium saturated which is saturated with nofluids. They have used uniform flux boundary conditions. According to a recent paper of Servati et al. [22], the metal porous medium owing to its high thermal conductivity, high specific surface area and good fluid mixing ability has been widely used for heat transfer enchantment in industries. Gao and Jin [23] presented the dynamics of oil—gas—water three-phase flow network mapping methods. They concluded that complex networks can be a potentially powerful tool for uncovering the nonlinear dynamics of oil—gas—water three-phase flow. Gao et al. [24] presented gas—liquid two-phase flow experiments in a small diameter pipe to measure local flow information from different flow patterns. They also presented a modality transition-based network for mapping the experimental multivariate measurements into a directed weighted complex network.

It is now well known that porous medium can be used to enhance the heat transfer rates. Transport phenomena in porous media have developed into a substantial subject area in its own right over the past two decades. Excellent summary of progress in this field has been given by recent books of Nield and Bejan [25], Vafai [26] where conductive, convective, radiative and coupled transport phenomena using various geometries, various boundary conditions and drag force approaches have been clearly illustrated. The modeling of porous media flow have received the interest of the researchers due to increasing demands on energy production, hydrocarbon extraction and chemical engineering packed bed systems. These areas have been addressed by Adler and Brenner [27]. Further applications include combustion systems [28] where porous media have demonstrated significant benefits compared with flame free combustion including enhanced burning rates, extended lean flammability limits and “green features” including marked reductions in emissions of pollutants. Other areas of application include electro-conductive polymer processing [29], and geophysics [30]. Very recently, Mahdi et al. [31] present a comprehensive review of nanofluid convective flow with heat transfer in porous media and explained the advantages of using porous media.

Probably, the idea of using a thermal convective boundary condition was first introduced by Aziz [32] to study the classical problem of forced convective flow over a flat plate. Following him many authors namely Makinde and Aziz [33], Merkin and Pop [34], Magyari [35] and Uddin et al. [36], Hayat et al. [37] to mention just a few of them, used this boundary condition for different boundary layer problems. Most of the researchers use thermal convective boundary conditions where heat transfer coefficient is function of axial distance. However, as pointed out by Pantokratoras [38], there might be no physical situation where heat transfer coefficient varies with the axial distance. Merkin et al. [39] investigated the mixed convection on a vertical surface in a Darcy porous medium with a constant convective boundary condition. Pantokratoras [40] reported mixed convection in a Darcy—Brinkman porous medium with a constant convective thermal boundary condition based on the cited literature. It would seem that mixed convective g-jitter flow in porous media with constant thermal and mass convective boundary conditions has not communicated in the literature which motivates the present analysis.

Based on the cited literature, it seems that, no studies of flow, heat and nanoparticle volume fraction, with constant convective thermal and mass boundary conditions effect in porous media have been communicated in the literature, which motivates our present study. Thus far to the best of our knowledge no research has been reported on the boundary layer flow of g-jitter induced mixed convective flow of nanofluids in a past a vertical surface embedded in porous media subject to both thermal and mass convective boundary conditions. The present paper in an extension of a recent paper of Uddin et al. [41] to Buongiorno-Darcy porous medium model and incorporation of constant convective thermal and mass convective boundary conditions. The governing conservation equations are converted to non-similar equations using relevant transformations. An implicit finite difference method has been used to solve the problem numerically. Comparison of our results with published paper is achieved for special case. The effect of emerging thermophysical parameters on the dimensionless velocity, temperature, nanoparticles volume fraction, friction factor, heat transfer rates and mass transfer rates are illustrated via figures.

### Description and Formulation of the Governing Equations

**Fig 1:**
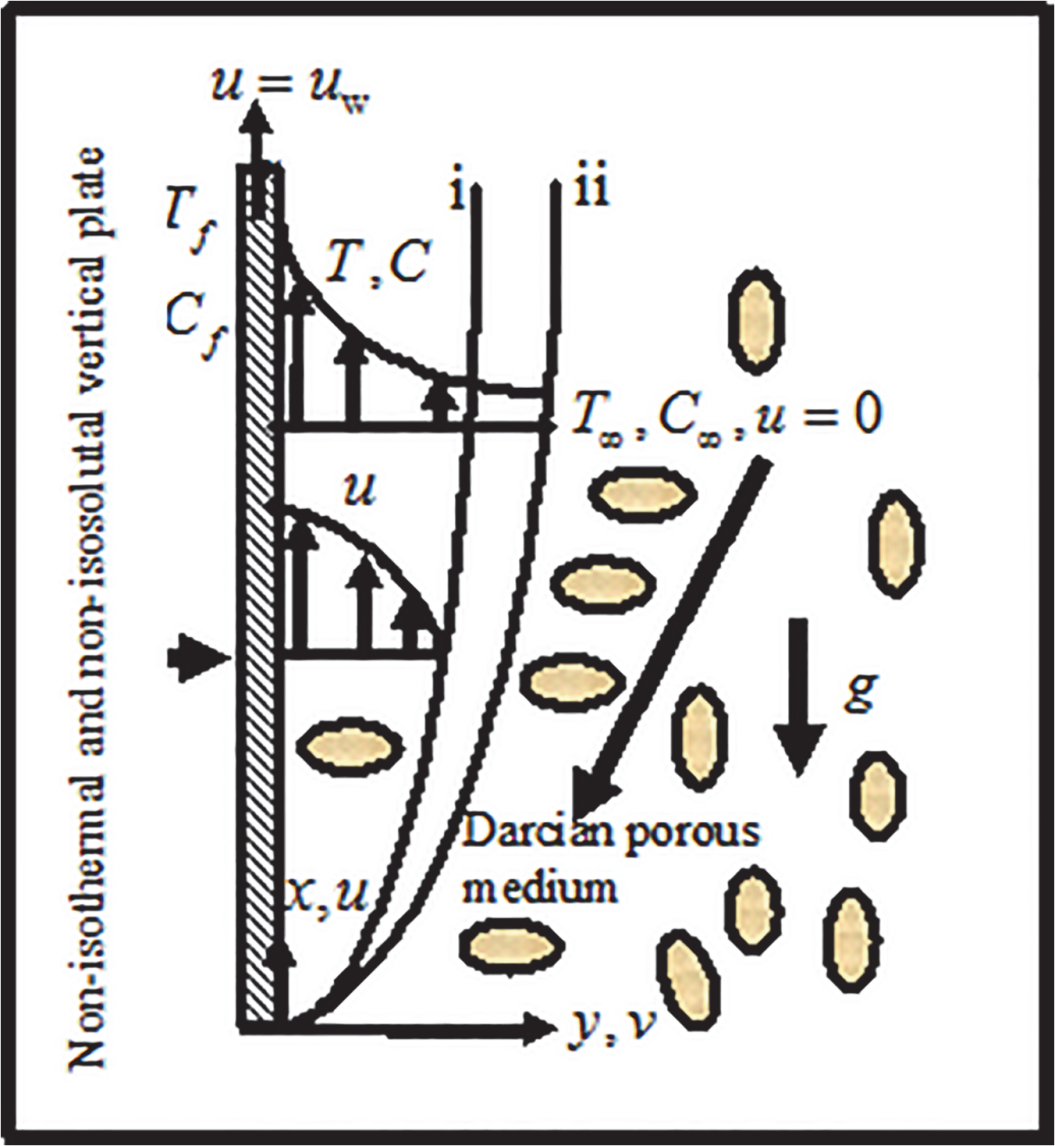
Geometry of the flow model and coordinate system.

Consider the 2-D laminar boundary layer flow of viscous incompressible nanofluids past a solid plate which is moving with a velocity $u_{w}=\left(\frac{U_{r}}{L}\right)x$ in the clam free stream. Here *U*_*r*_ is the characteristic velocity and *L* is the characteristic length. The plate surface is subjected to constant thermal and mass convective boundary conditions. The effect of g-jitter is induced by mixed convective flow of a nanofluid past the plate. The gravity acceleration is given by $g^{*}(t)=g_{0}\left[1+\varepsilon \cos\left(\pi \omega \;t\right)\right]\vec{K}$ where *g*_0_ is the time-averaged value of the gravitational acceleration. g*(*t*) acting along the direction on the unit vector$\vec{K}$, which is oriented in the upward direction, *ε* is a scaling parameter, which yields the magnitude of the gravity modulation relative to *g*_0_, *t* is the time and *ω* is the frequency of oscillation of the g-jitter driven flow (Sharidan et al. [8]). If *ε* << 1 then the forcing may be seen as a perturbation of the mean gravity. It is considered that the left of the plate is heated by the convection from the hot fluid of temperature *T*_*f*_(>*T*_*w*_>*T*_*∞*_) which yields a constant heat transfer coefficient *h*_*f*_. Consequently a thermal convective boundary condition arises. It is also considered that the concentration of the nanoparticle in the left of the plate *C*_*f*_(>*C*_*w*_>*C*_*∞*_) is higher than that of the plate concentration *C*_*w*_ and free stream concentration *C*_*∞*_ which gives a constant mass transfer coefficient *h*_*m*_. As a result a mass convective boundary condition arises (Uddin et al. [41]). The model problem under consideration along with the coordinate system is shown in Fig 1.

The governing equations can be written in terms of dimensional forms, extending the formulations of Buongiorno [14]

$$\frac{\partial u}{\partial x}+\frac{\partial \text{v}}{\partial y}=0,$$ 1

$$\frac{\partial u}{\partial t}+u\frac{\partial u}{\partial x}+\text{v}\frac{\partial u}{\partial y}=\nu \frac{\partial^{2}u}{\partial y^{2}}-\frac{\nu }{k_{p}}u+g*(t)\beta_{T}\left(T-T_{\infty }\right)\left(\frac{x}{L}\right)+g*(t)\beta_{C}\left(C-C_{\infty }\right)\left(\frac{x}{L}\right),$$ 2

$$\frac{\partial T}{\partial t}+u\frac{\partial T}{\partial x}+\text{v}\frac{\partial T}{\partial y}=\alpha \frac{\partial^{2}T}{\partial y^{2}}+\tau \left[D_{B}\frac{\partial \phi }{\partial y}\frac{\partial T}{\partial y}+\frac{D_{T}}{T_{\infty }}{\left(\frac{\partial T}{\partial y}\right)}^{2}\right],$$ 3

$$\frac{\partial C}{\partial t}+u\frac{\partial C}{\partial x}+\text{v}\frac{\partial C}{\partial y}=D_{B}\frac{\partial^{2}C}{\partial y^{2}}+\frac{D_{T}}{T_{\infty }}\frac{\partial^{2}T}{\partial y^{2}}.$$ 4

Following Uddin et al. [41] and Dayan et al. [42], the initial and boundary conditions for the present problem are

$$\begin{array}{l} t<0,u=\text{v}=0\;\text{any}\;x,\;y\\ \begin{array}{l} t>0,u=u_{w},\text{v}=0,-k\frac{\partial T}{\partial y}=h_{f}(T_{f}-T),-D_{B}\frac{\partial C}{\partial y}=h_{m}\left(C_{f}-C\right)\;\text{at}\;y=0,\\ u\to 0,T\to T_{\infty },C\to C_{\infty }\;\text{as}\;y\to \infty . \end{array} \end{array}$$ 5

Here $\alpha =\frac{k}{{\left(\rho c\right)}_{f}}$is the thermal diffusivity of the fluid, $\tau =\frac{{\left(\rho c\right)}_{p}}{{\left(\rho c\right)}_{f}}$is the ratio of heat capacity of the nanoparticle and fluid, is the permeability of the medium, (*u*,*v*) are the Darcian velocity components along the *x* and *y*-axes, *u*_*w*_ is the velocity of the plate, *v* is the kinematic viscosity of the fluid, *D*_*B*_ is the Brownian diffusion coefficient, *D*_*T*_ is the thermophoretic diffusion coefficient, *β*_*T*_ is the coefficient of thermal expansion, *β*_*C*_ is the coefficient of mass expansion, *k*_*p*_ is the permeability of the porous media. The last two terms of Eq (2) are due to thermal and concentration buoyancy effects which are due to the temperature and concentration of nanoparticle differences. These two terms originate from well-known Boussinesq approximation. In order to reduce the number of the dependent variables as well as number of equations, we use stream function *ψ* defined by$u=\frac{\partial \psi }{\partial y},v=-\frac{\partial \psi }{\partial x}$. Note that *ψ* satisfies equation of continuity automatically. Now, introducing the following transformations (Uddin et al. [41])

$$\begin{array}{l} \tau =\omega t,\eta =\frac{y}{\sqrt{k_{p}}},u=\frac{U_{r}x}{L}\frac{\partial f\left(\eta ,\tau \right)}{\partial \eta },\text{v}=-\frac{U_{r}x}{L}\sqrt{k_{p}}f\left(\eta ,\tau \right),\\ \theta =\frac{T-T_{\infty }}{T_{f}-T_{\infty }}=\theta \left(\eta ,\tau \right),\phi =\frac{C-C_{\infty }}{C_{f}-C_{\infty }}=\phi \left(\eta ,\tau \right),g\left(\tau \right)=\frac{g^{*}\left(t\right)}{g_{0}}. \end{array}$$ 6

Substitution of transformation variables (6) into Eqs (2)–(4), yield

1Da Re∂3f∂η3+f∂2f∂η2−(∂f∂η)2−1Da Re∂f∂η+(1+εcosπτ)λ[θ+Nrϕ]=Ω∂2f∂τ∂η, 7

1Da RePr∂2θ∂η2+f∂θ∂η+Nb∂θ∂η∂ϕ∂η+Nt(∂θ∂η)2=Ω∂θ∂τ, 8

1Da ReSc∂2ϕ∂η2+f∂ϕ∂η+NbNt∂2θ∂η2=Ω∂ϕ∂τ. 9

The boundary conditions (5) become

$$\begin{array}{l} \frac{\partial f}{\partial \eta }\left(\tau ,0\right)=1,f\left(\tau ,0\right)=0,\frac{\partial \theta }{\partial \eta }\left(\tau ,0\right)=-Nc\left[1-\theta \left(\tau ,0\right)\right],\\ \frac{\partial \phi }{\partial \eta }\left(\tau ,0\right)=-Nd\left[1-\phi \left(\tau ,0\right)\right],\frac{\partial f}{\partial \eta }\left(\tau ,\infty \right)=\theta \left(\tau ,\infty \right)=\phi \left(\tau ,\infty \right)=0. \end{array}$$ 10

The dimensionless parameters are: $\mathrm{Pr}=\frac{\nu }{\alpha }$ is the Prandtl number, $\Omega =\frac{\omega L}{U_{r}^{}}$ is the non-dimensional frequency, *ε* is the amplitude of the modulation, $\lambda =\frac{g_{0}\beta_{T0}\Delta TL^{3}}{U_{r}^{2}}$ is the mixed convection parameter, $Nr=\frac{\beta_{C}\Delta C}{\beta_{T}\Delta T}$ is the buoyancy ratio parameter, $Da=\frac{k_{p}}{L^{2}}$ is the Darcy number, Re=UrLν is the Reynolds number, $Nt=\frac{\tau D_{T}\left(T_{f}-T_{\infty }\right)}{k_{p}U_{r}T_{\infty }}$ is the thermophoresis parameter, $Nb=\frac{\tau D_{B}\left(C_{f}-C_{\infty }\right)L}{k_{p}U_{r}}$ is the Brownian motion parameter, $Sc=\frac{\nu }{D_{B}}$ is the Schmidt number, $Nd=\frac{h_{m}\sqrt{k_{p}}}{D_{B}}$ is the convection-diffusion parameter and $Nc=\frac{h_{f}\sqrt{k_{p}}}{k}$ is the convection-conduction parameter.

### Quantities of Physical Interest

The quantities of engineering interest, in this study, are the local skin friction factor *C*_*fx*_, the local Nusselt number *Nu*_*x*_, the local Sherwood number *Sh*_*x*_ can be found from the following definition

$$C_{fx}=\frac{\tau_{w}}{\rho u_{w}^{2}},Nu_{\bar{x}}=\frac{xq_{w}}{k_{f}\left(T_{f}-T_{\infty }\right)},Sh_{\bar{x}}=\frac{xm_{w}}{D_{B}\left(C_{f}-C_{\infty }\right)},$$ 11

where *τ*_*w*_,*q*_*w*_, *m*_*w*_ are shear stress, the wall heat flux, the wall mass flux and are defined as

$$\tau_{w}=-\mu {\left(\frac{\partial u}{\partial y}\right)}_{y=0},q_{w}=-k{\left(\frac{\partial T}{\partial y}\right)}_{y=0},m_{w}=-D_{B}{\left(\frac{\partial C}{\partial y}\right)}_{y=0}.$$ 12

Using Eqs (6) and (12), we have from Eq (11)

Rex1/2Cfx=∂2f∂η2(τ,0),Rex1/2Nux=−∂θ∂η(τ,0),Shx1/2Nux=−∂ϕ∂η(τ,0), 13

where Rex=uwxν is the local Reynolds number.

### Comparison of Our Results with Literature

In the absence of the nanoparticle equation, it is interesting to note that if we put *Da* = 1, *λ* = Ω = 0, *Nb* = *Nt* → 0, *Nc* = *Nd* → ∞, in Eqs (7) and (8), we have the same eqns. as derived by Dayyan et al. [42] when we put *n = 0* in their paper. Hence we are confident about our analysis. Now, before using the present numerical solution technique to the present problem, it was used to a case considered by Dayyan et al. [42] in order to justify its correctness. The results are exhibited in Tables 1 and 2. A good agreement is found.

**Table 1:** Comparison of Skin-friction factor (−*f*″(0)) for several Reynolds number when Da = 1, *a* = *M* = 0, *R* → ∞.

Re	Dayyan et al. [42]		Present
	RK	HAM	Finite difference
1	1.4242	1.4198	1.4198
1.5	1.5811	1.5799	1.5808
2	1.7320	1.7234	1.7319
5	2.4494	2.4394	2.4492

**Table 2:** Comparison between RKF45, HAM and RK for the values of heat transfer rate (−*θ*′(0)) for several values of Reynolds number when *Nc* = *Nd* = *R* → ∞, Pr = *Da* = 1.

Re	Dayyan et al. [42]		Present result
	RK	HAM	Finite difference
1	0.5033	0.5030	0.5038
1.5	0.6422	0.6456	0.6430
2	0.7592	0.7518	0.7539
5	1.2576	1.2636	1.2551

## Results and Discussions

**Fig 2:**
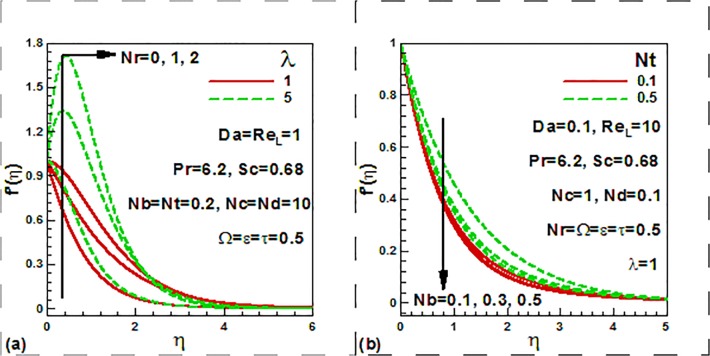
Variation of dimensionless velocity with (a) mixed convection and buoyancy ratio and (b) nanofluid parameters.

Eqs (7)–(9) with boundary conditions (10) were solved numerically by using by an implicit finite difference method with quasi-linearization technique for various values of the controlling parameters. Fig 2(A) and 2(B) show the variation of the dimensionless velocity with the mixed convection and buoyancy ratio, thermophoresis and Brownian motion parameters. It is noticed from Fig 2A that the mixed convection parameter increases the velocity both in the presence and absence of the buoyancy ratio. Fig 2B shows that the dimensionless velocity decreases with an increase in the Brownian motion parameter and the opposite trend is noticed for the case of the thermophoresis parameter.

**Fig 3:**
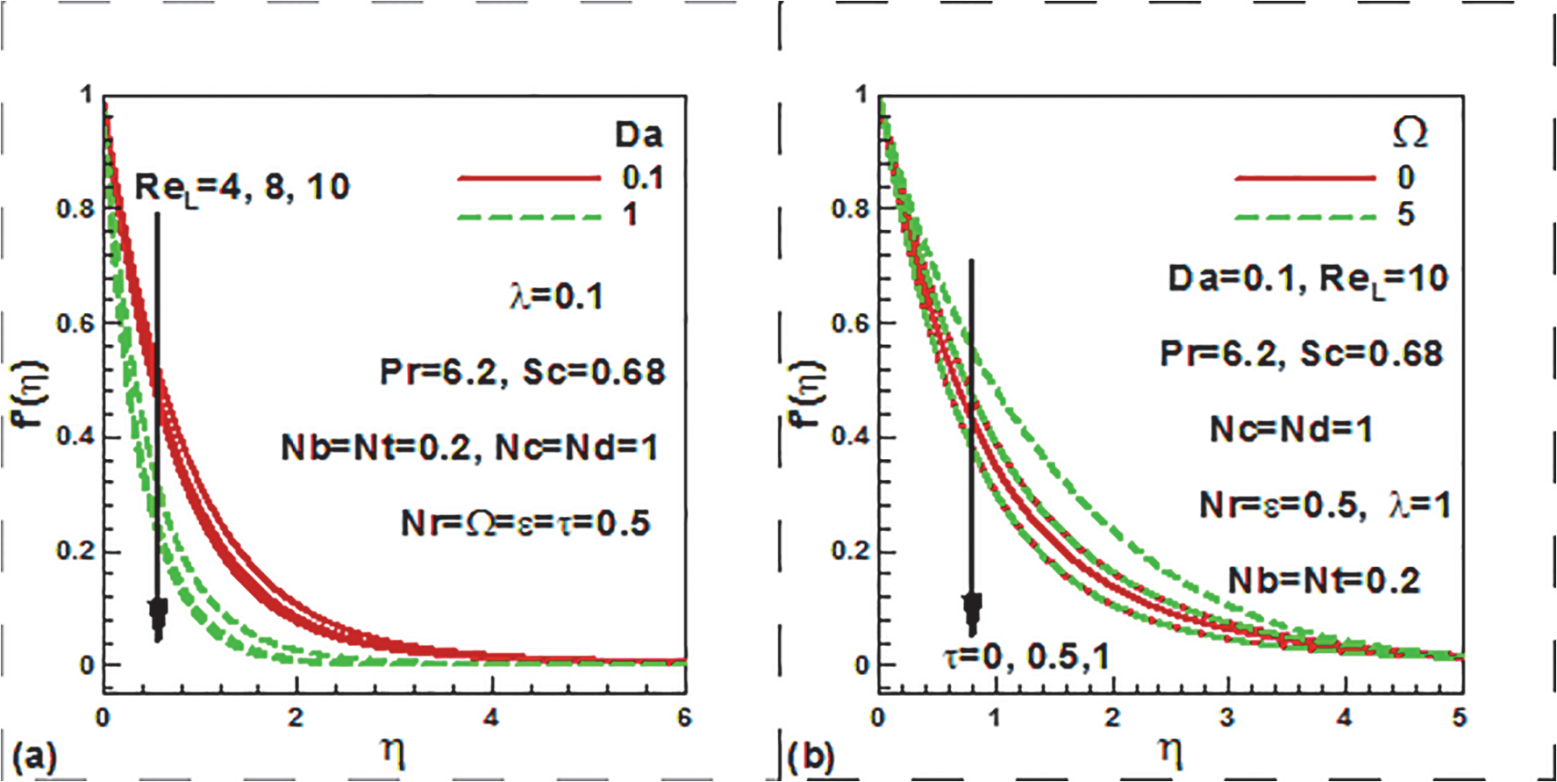
Variation of dimensionless velocity with (a) Darcy and Rayleigh numbers and (b) dimensionless time and frequency parameters.

Fig 3 shows the variation of the dimensionless velocity with the Darcy number, Reynolds number, dimensionless time and frequency parameters It is noticed from Fig 3(A) that with an increase in the Reynolds number, the dimensionless velocity reduces. It is also observed that the velocity reduces as Darcy number increases. In the transformed momentum Eq (7), the term−1Da Re∂f∂η, represents the porous medium drag force, based on the Darcy law. This term is inversely proportional to permeability of the porous material. Enhancing Da will therefore enhance permeability to reduce the impedance from porous media fibers to the fluid, thereby decelerating the flow. This is in agreement with the trend shown in **Fig** 3A. Fig 3B shows that velocity decreases with an increase in the dimensionless time. The opposite trend is noticed in the case of dimensionless frequency parameter.

**Fig 4:**
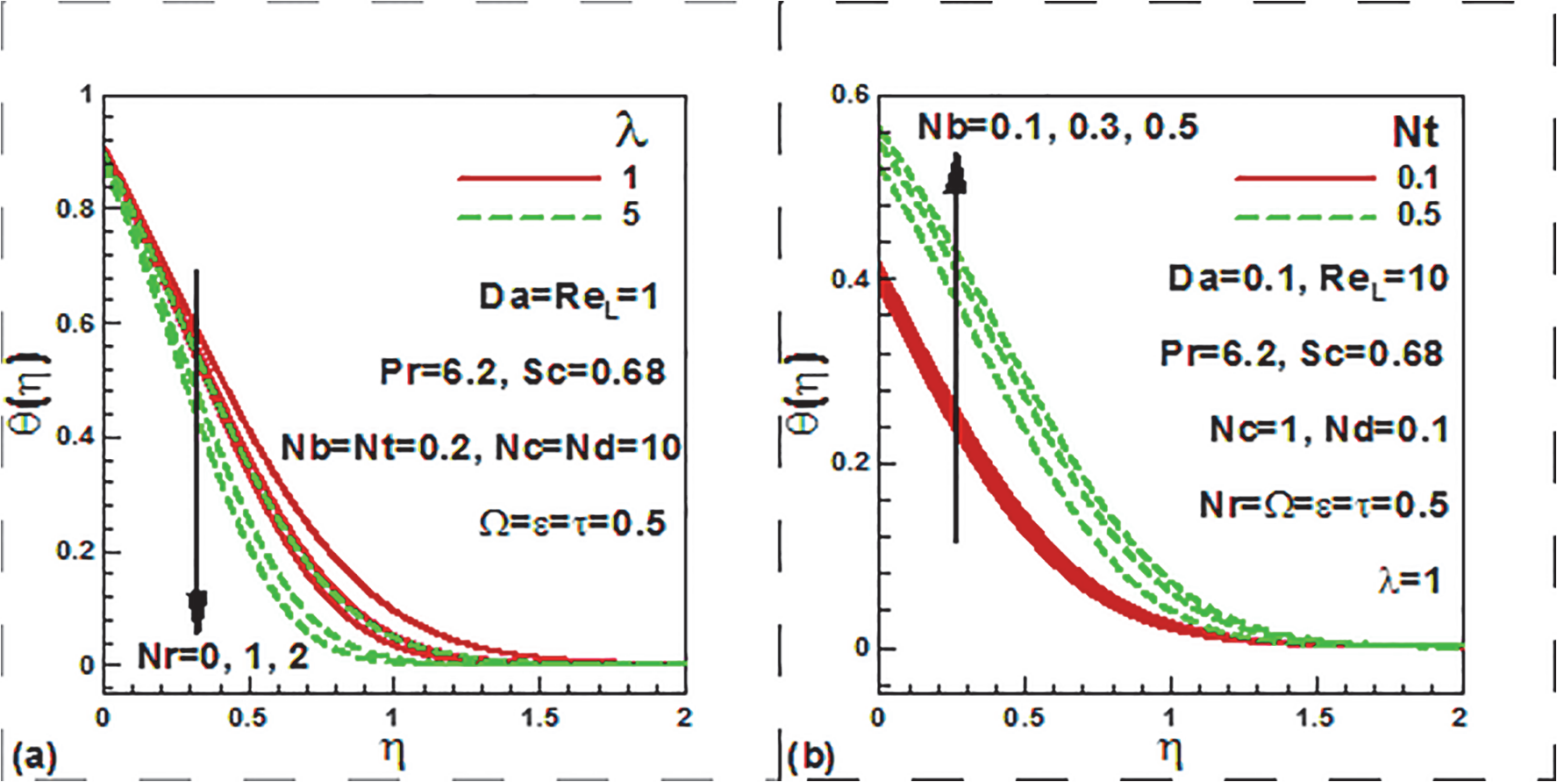
Variation of dimensionless temperature with (a) mixed convection and buoyancy ratio and (b) nanofluid parameters.

Fig 4 presents the influence of the mixed convection parameter, buoyancy ratio, thermophoresis and Brownian motion parameters on the nondimensional temperature profiles. It is noticed from Fig 4A that the mixed convection parameter decreases the velocity both in the presence and absence of the buoyancy ratio. It is further noticed that temperature is decreases with the increase of the buoyancy ratio parameter. The temperature at the wall as well as in the thermal boundary layer is increased with an increase in both the Brownian motion and thermophoresis parameters (Fig 4B). Theoretically smaller nanoparticles possess higher *Nb* values, which aid in thermal diffusion in the boundary layer via enhanced thermal conduction. On the other hand larger nanoparticle shows lower *Nb* values and this reduces thermal conduction. Higher *Nb* values will conversely stifle the diffusion of nanoparticle away from the surface into the fluid regime lead to reduce in nanoparticle concentration values in the boundary layer. The distribution of nanoparticle in the boundary layer regime can therefore be regulated via the Brownian motion mechanism (higher *Nb* values) and cooling of the regime can also be achieved via smaller *Nb* values. Thermal enhancement is obtained with higher *Nb* values. Larger thermal boundary layer thickness is produced with higher *Nb* values whereas larger concentration boundary layer thickness is obtained with lower *Nb* values.

**Fig 5:**
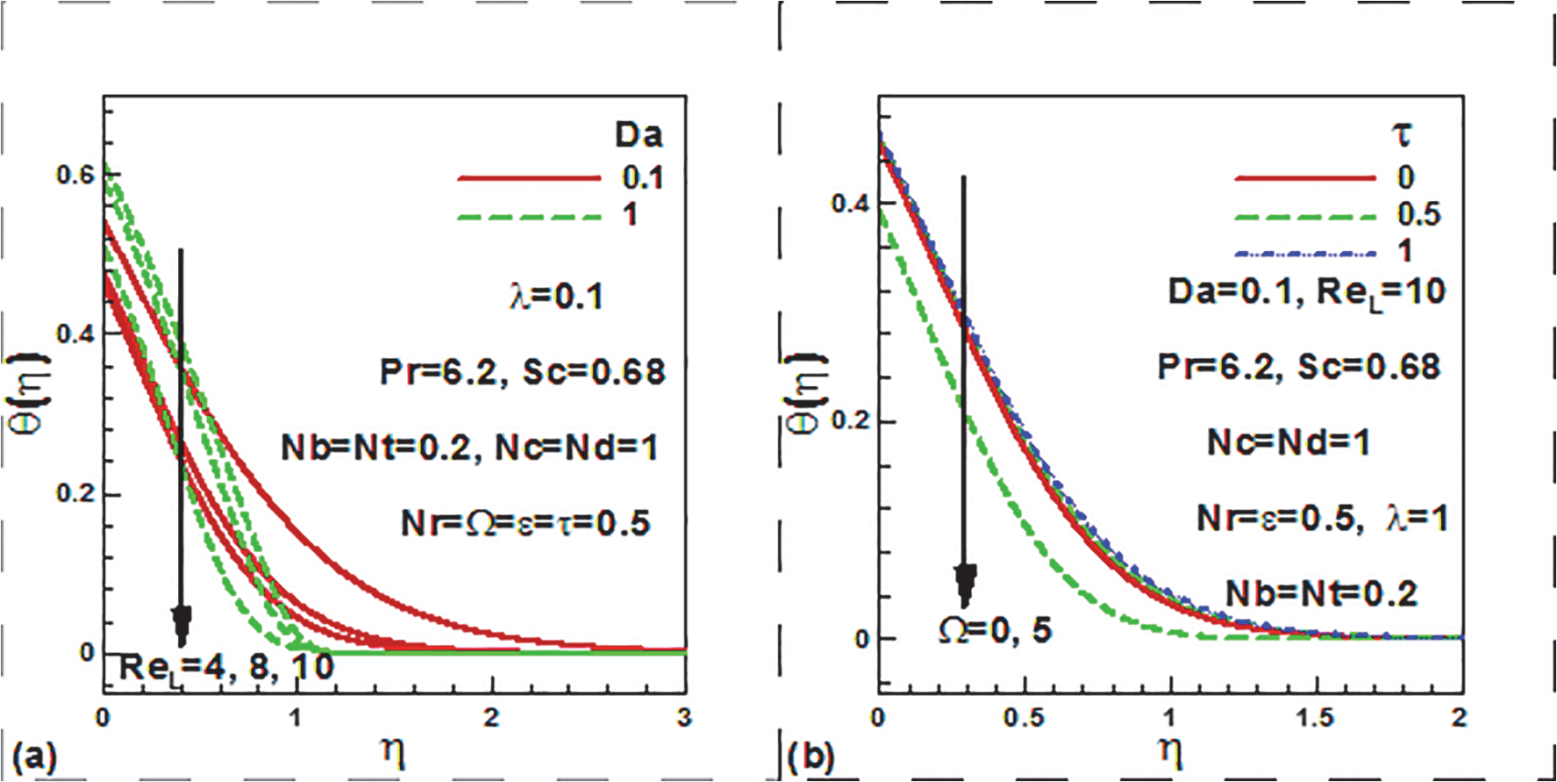
Variation of dimensionless temperature with (a) Darcy and Rayleigh numbers and (b) dimensionless time and frequency parameters.

**Fig 6:**
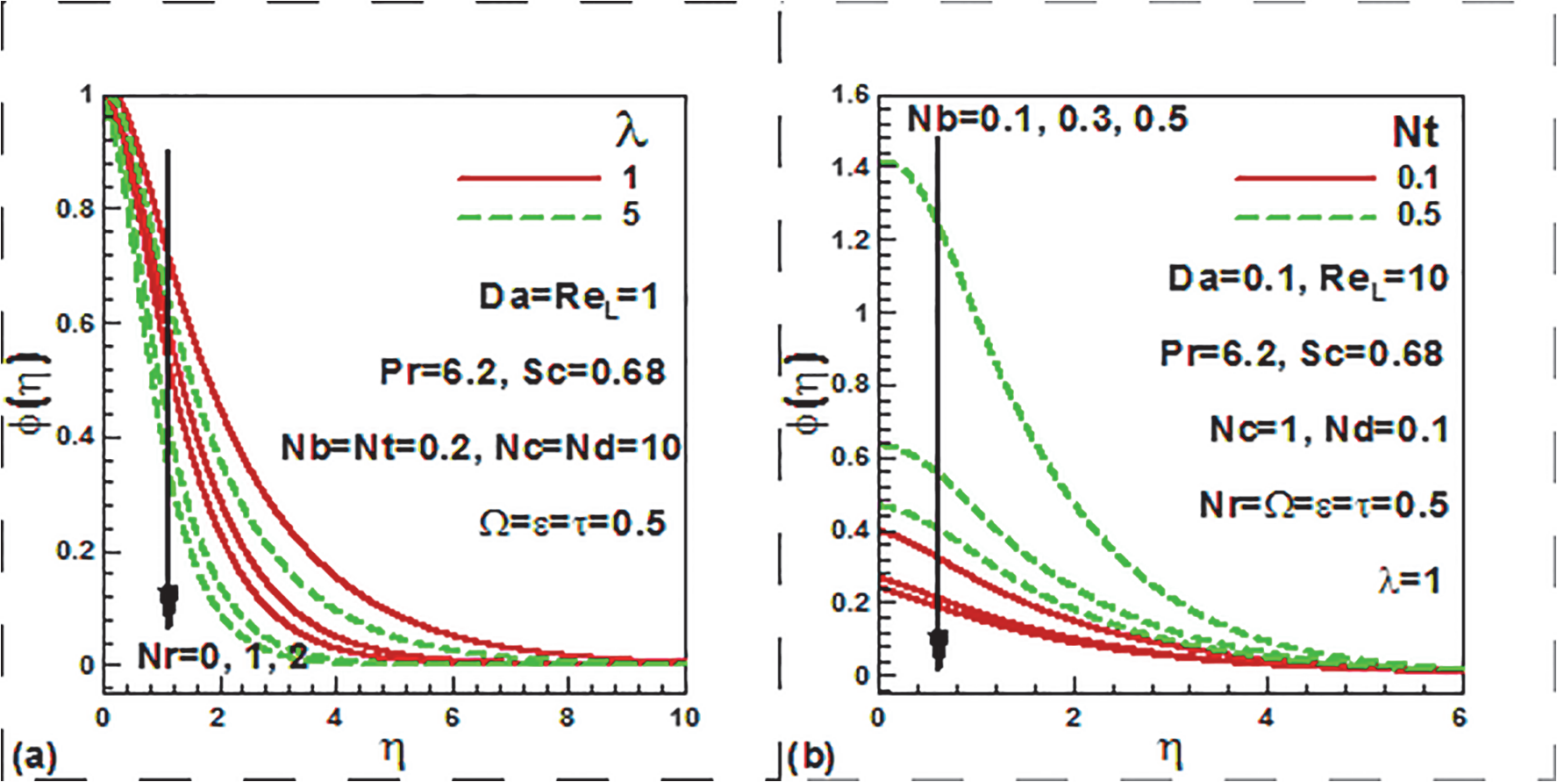
Variation of dimensionless concentration with (a) mixed convection and buoyancy ratio and (b) nanofluid parameters.

Fig 5A and 5B illustrates the distributions of the dimensionless temperature with a variation in the Darcy number, Reynolds number, dimensionless time and frequency parameters. It is observed from Fig 5A that with the increase of the Reynolds number, the dimensionless temperature reduces. It is also observed that the temperature increases as Darcy number increases near the wall. It is further noticed that temperature is reduced inside the thermal boundary layer. In fact, higher Darcy number implies a higher permeabiity in the porous medium. This corresponds to a decrease in presence of solid fibers and a reduction in thermal conduction heat transfer in the medium. Increasing Da values leads to decrease in temperatures in the regime, as clearly observed in Fig 5A. This will be accompanied by a decrease in thermal boundary layer thickness. Fig 5B shows that temperature decreases with the increase of the dimensionless frequency parameter for both steady and unsteady case. Fig 6 illustrates the influence of the mixed convection parameter, buoyancy ratio, thermophoresis and Brownian motion parameters on the dimensionless concentration profiles.

The concentration decreases with the increase of both the mixed convection and buoyancy ratio parameters (Fig 6A). Concentration is decreased as the Brownian motion parameter increases and the opposite behavior is noticed in the case of thermophoresis parameter (Fig 6B).

**Fig 7:**
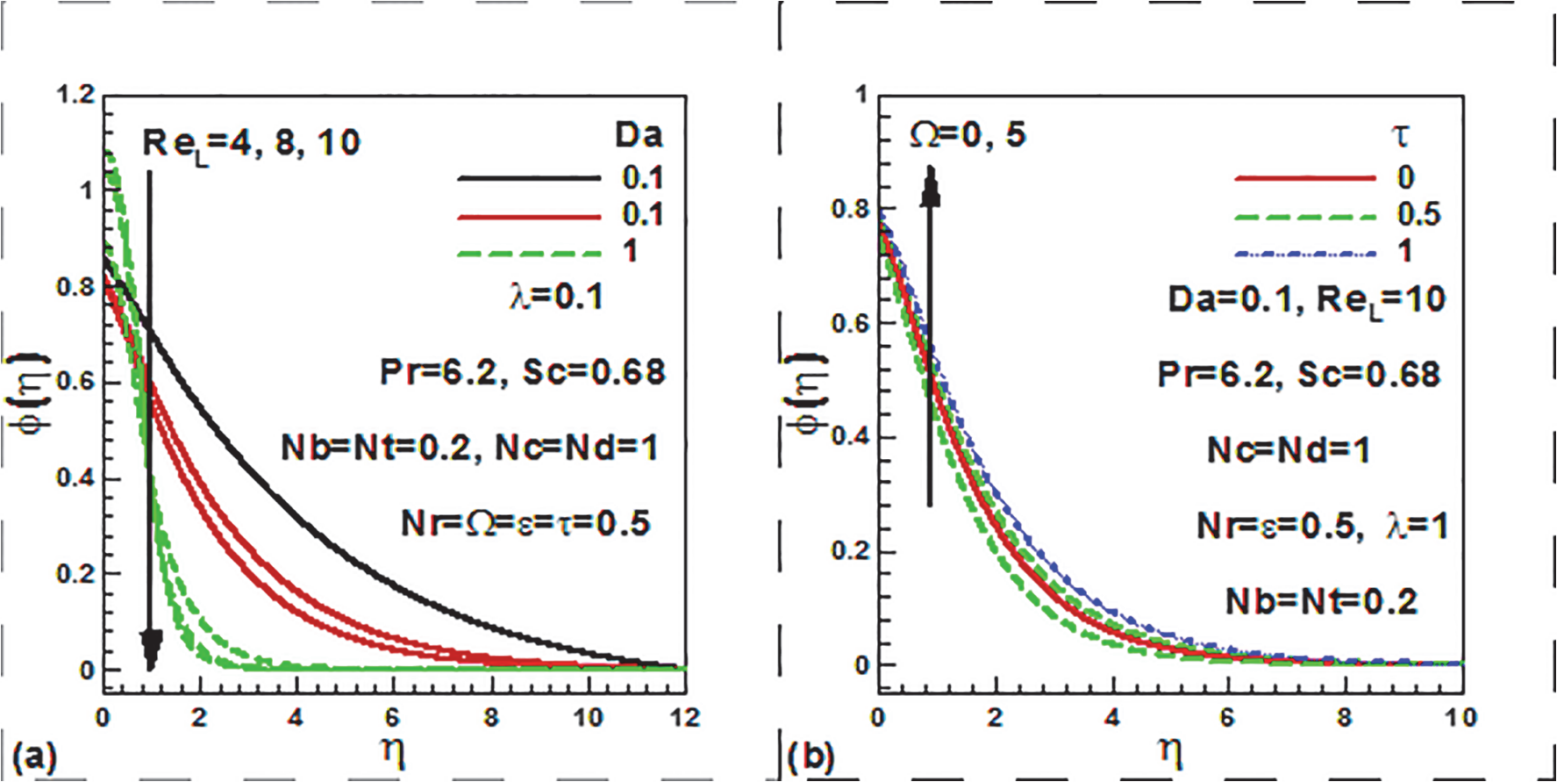
Variation of dimensionless concentration with (a) Darcy and Rayleigh numbers and (b) dimensionless time and frequency parameters.

Fig 7 displays the influence of the Reynolds number, Darcy number, dimensionless time and frequency parameter on the dimension less nanoparticle volume fraction profiles. It is found that both the Reynolds number and Darcy number reduce the concentration (Fig 7A). Temperature is increased with the increase of the frequency parameter for both steady and unsteady case (Fig 7B).

**Fig 8:**
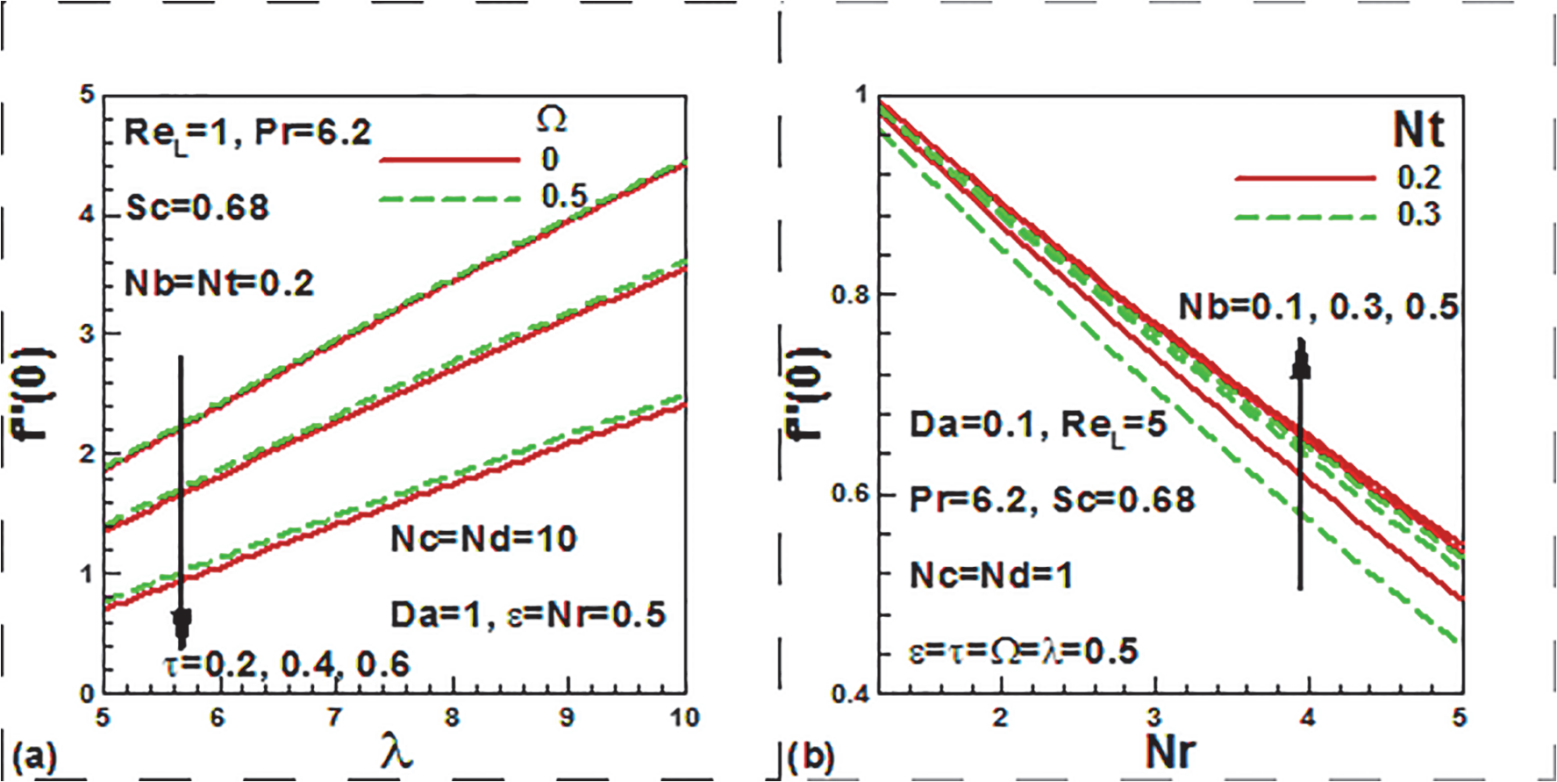
Effects of dimensionless time, frequency and nanofluid parameters on skin friction.

We now focus on the effect of the entering parameters on the quantities of practical interest. Fig 8A and 8B show the combined effects of the dimensionless time, frequency, thermophoresis, Brownian motion, buoyancy ratio, and mixed convection parameters on the skin friction factor. With increasing time, skin friction factor is strongly decreased. With the increase of mixed convection parameter and dimensionless frequency parameter, skin friction factor is strongly increased (Fig 8A). From Fig 8B, it is observed that with the increase of the Brownian motion, skin friction factor is strongly increased opposite trend of skin friction is noticed with the increase of buoyancy ratio, and thermophoresis parameters. The combined effects of the dimensionless time, frequency, thermophoresis parameter, Brownian motion, buoyancy ratio, and mixed convection parameters on the heat transfer rates.

**Fig 9:**
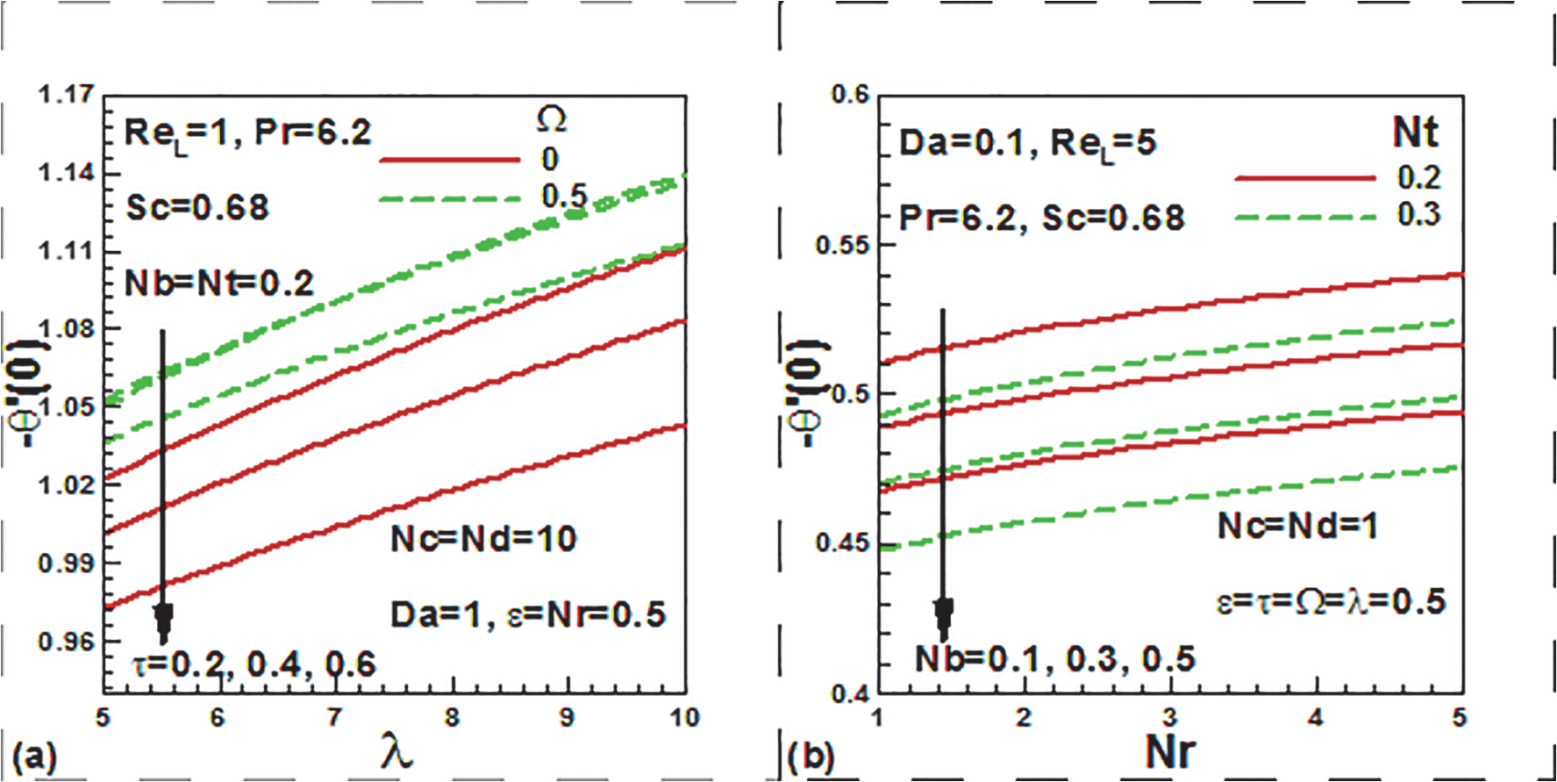
Effects of dimensionless time, frequency and nanofluid parameters on dimensionless heat transfer rate.

It is noticed that with time, the heat transfer rates is decreased. With the increase of mixed convection parameter and dimensionless frequency parameter, heat transfer rate is increased (Fig 9A). From Fig 9(B), it is found that with the increase of the Brownian motion and themophoresis parameters, the heat transfer rates is reduced opposite trend is noticed with the increase of buoyancy ratio parameters.

**Fig 10:**
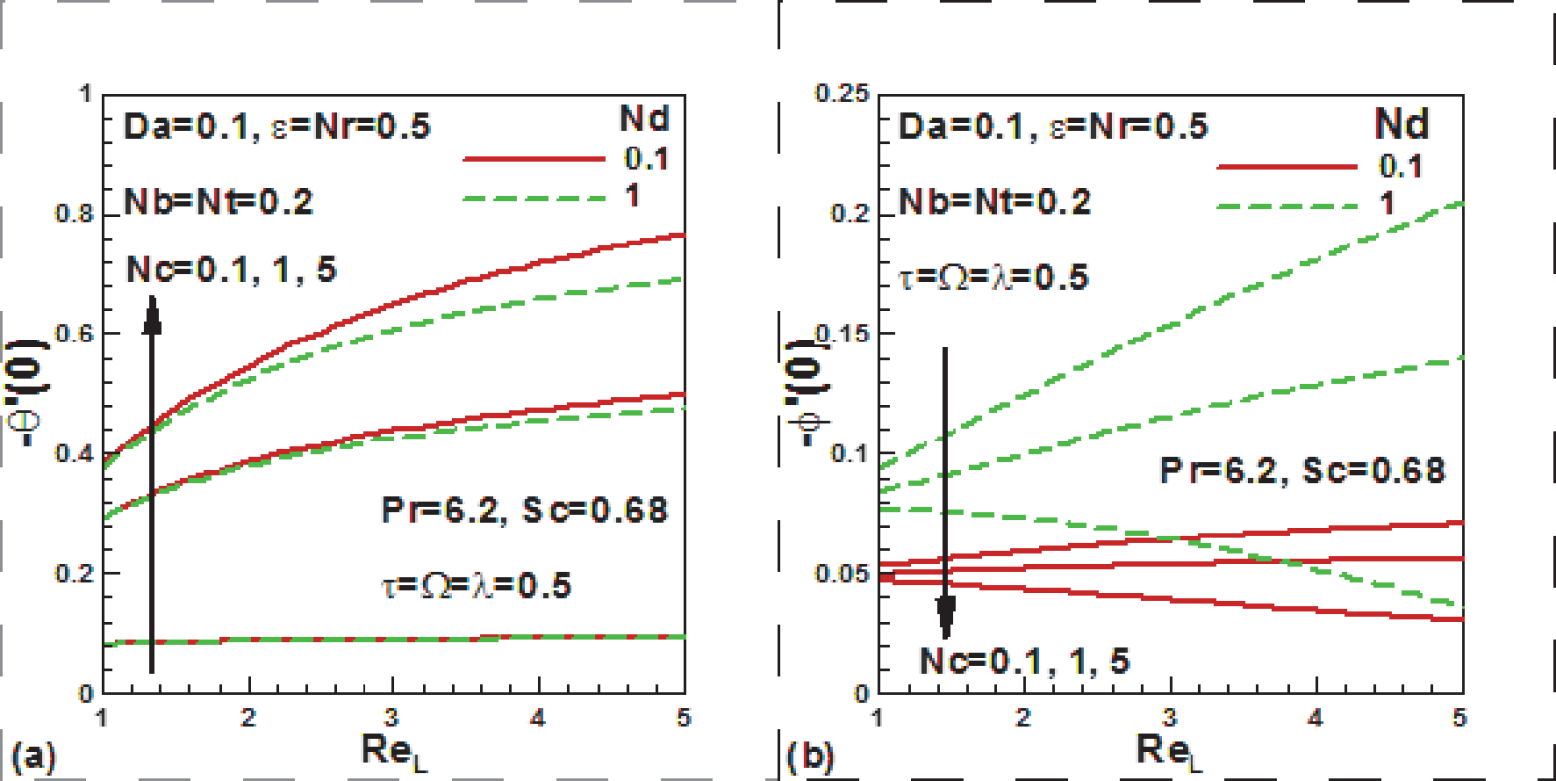
Effects of Reynolds number with dimensionless convection-diffusion and conduction parameters on dimensionless heat and mass transfer rate.

Fig 10A and 10B show the effects of the Reynolds number, convection-diffusion and convection-conduction parameters on dimensionless heat transfer rates (Fig 10A) and mass transfer rates (Fig 10B). The convection-conduction parameter *Nc* is basically a thermal Biot number which is the ratio of the internal thermal resistance of a solid to the boundary layer thermal resistance. When *Nc = 0* (insulated plate), there will be no heat transfer from the left side to right side of the plate. The convection-diffusion parameter *Nd* is similarly effectively a solutal Biot number. An inspection of Fig 10A reveals that heat transfer rates increases with the increase of the convection-diffusion parameter and Reynolds number. The reverse trend is noticed in the case of convection-diffusion parameter. It is found from Fig 10B that mass transfer rates decreases with the increase of the convection-conduction parameter, reverse trends is noticed in the case of convection-diffusion parameter.

**Fig 11:**
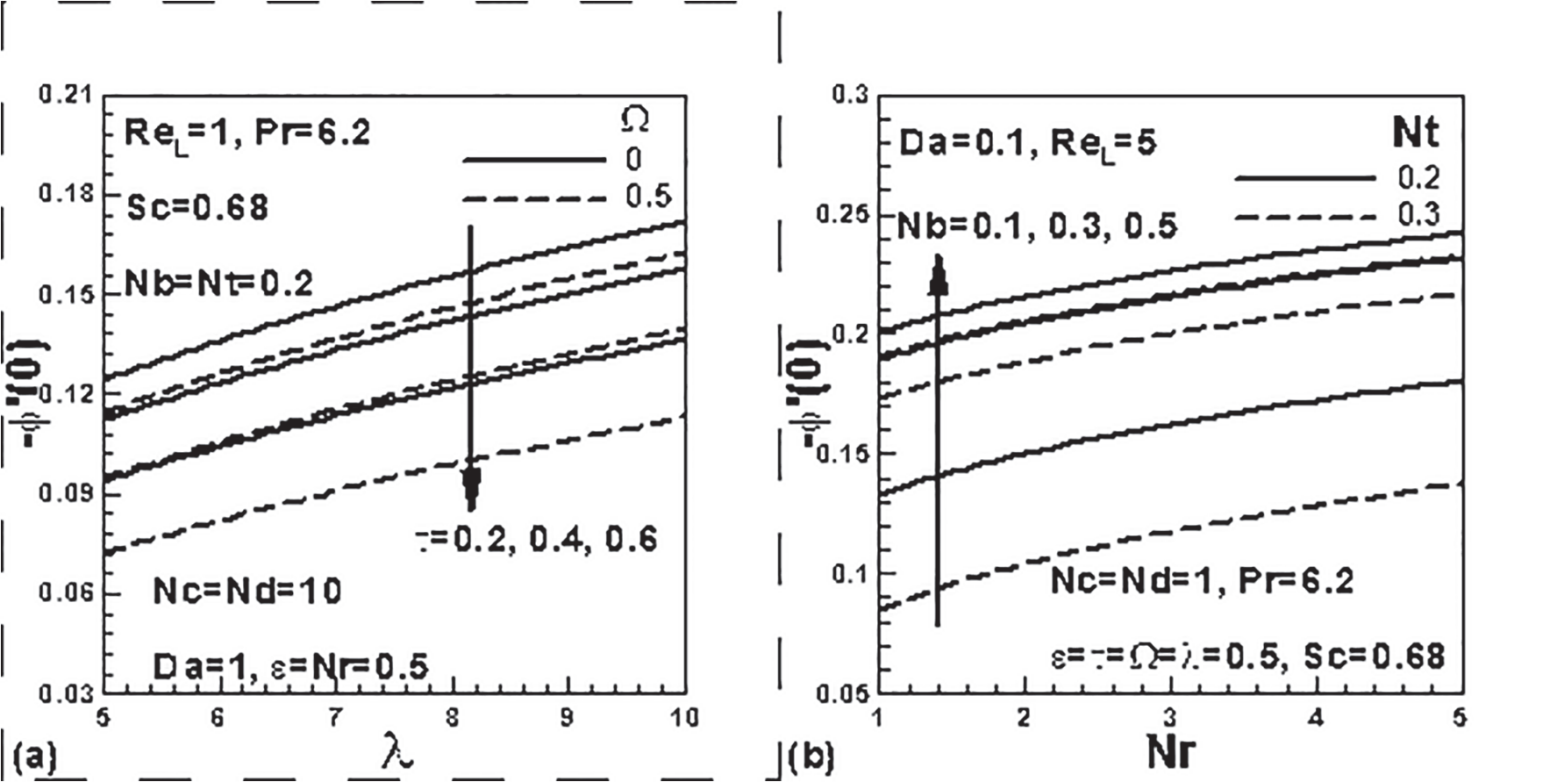
Effects of dimensionless time, frequency and nanofluid parameters on dimensionless mass transfer rate.

Fig 11A and 11B show the effects of dimensionless time, frequency and nanofluid parameters on dimensionless mass transfer rates. From an inspection of Fig 11A, it is noticed that mass transfer rates decreases with the increases of the time and frequency parameter and the reverse trend is noticed in the case of the mixed convection parameter. From Fig 11B, it is noticed that mass transfer rates increases with both the Brownian motion and buoyancy ratio parameters whereas it is decreased with the thermophoresis parameter.

## Conclusion

In this paper, the two-dimensional g-jitter mixed convective boundary layer flow of water-based nanofluids past a moving plate in a Darcian porous medium is investigated by combined non-similar and numerical solution techniques. The main findings are given below.

1. The skin friction decreases with time, buoyancy ratio and thermophoresis parameters whilst it increases with frequency parameter, mixed convection parameter and Brownian motion parameters.
2. The heat transfer rates decreases with time, Brownian motion parameter, thermophoresis and diffusion-convection parameters whilst it increases with Reynolds number, frequency, mixed convection, buoyancy ratio and conduction-convection, parameters.
3. The mass transfer rates decreases with time, frequency parameter, thermophoresis, conduction-convection parameters whilst it increases with mixed convection, buoyancy ratio, diffusion-convection and Brownian motion parameters
